# Operating room extubation: experience from a public Brazilian pediatric cardiovascular center

**DOI:** 10.1590/1984-0462/2026/44/2026039

**Published:** 2026-11-13

**Authors:** Thayane Carvalho Vieira, Beatriz Correa Tinoco, Gabriella Eduarda Lins de Souza, Luana Carvalho Vieira, Flávia Xavier de Oliveira, Fellipe Allevato Martins da Silva, Roberta da Silva Teixeira

**Affiliations:** a Instituto Nacional de Cardiologia, Rio de Janeiro, RJ, Brazil.

**Keywords:** Extubation, Heart defects, Congenital, Operating rooms, Thoracic durgery, Quality indicators, Health care, Extubação, Cardiopatias congênitas, Salas cirúrgicas, Cirurgia torácica, Indicadores de qualidade em assistência à saúde

## Abstract

**Objective:**

To investigate the incidence and success of intentional postoperative operating room extubation after elective congenital cardiac surgery at a Brazilian federal referral center and identify factors associated with non-extubation and extubation failure.

**Methods:**

This historical cohort study was conducted in a pediatric and neonatal cardiac intensive care unit over five years. Patients aged 0–18 years with congenital heart disease who underwent elective corrective or palliative cardiac surgery without preoperative mechanical ventilation were included. The primary outcome was operating room extubation, and the secondary outcome was extubation success, defined as no reintubation within 48 postoperative hours. Multivariable Poisson regression with robust variance identified factors independently associated with nonextubation and extubation failure (p<0.05).

**Results:**

Among 637 patients, 37% were extubated in the operating room, with a 98% success rate. They were older, had higher body weight, shorter noninvasive ventilation duration, shorter intensive care unit stays, and lower mortality. Increasing age reduced the risk of non-extubation. The need for noninvasive ventilation and longer intensive care unit stay were independently associated with non-extubation and extubation failure, although only four failures occurred.

**Conclusions:**

Operating room extubation was feasible in a substantial proportion of pediatric patients and achieved a high success rate, supporting its safety in selected cases. Greater surgical complexity, need for noninvasive ventilation, and longer intensive care unit stay increased the risk of non-extubation, whereas increasing age reduced this risk.

## RESUMO

**Objetivo:**

Investigar a incidência e o sucesso da extubação intencional em sala operatória após cirurgia cardíaca congênita eletiva em um centro federal brasileiro de referência e identificar fatores associados à não extubação e à falha de extubação.

**Métodos:**

Estudo de coorte histórica realizado em unidade de terapia intensiva cardíaca pediátrica e neonatal durante cinco anos. Foram incluídos pacientes de zero a 18 anos com cardiopatia congênita submetidos à cirurgia cardíaca corretiva ou paliativa, sem ventilação mecânica préoperatória. O desfecho primário foi a extubação em sala operatória, e o secundário, o sucesso da extubação. Regressão de Poisson multivariada com variância robusta identificou fatores associados à não extubação e à falha de extubação (p<0,05).

**Resultados:**

Entre 637 pacientes, 37% foram extubados na sala operatória, com 98% de sucesso. Esses pacientes eram mais velhos, tinham maior peso corporal, menor tempo de ventilação não invasiva, menor permanência na unidade de terapia intensiva e menor mortalidade. O aumento da idade reduziu o risco de não extubação, enquanto a categoria 4 do Risk Adjustment in Congenital Heart Surgery (RACHS-1) aumentou esse risco. Necessidade de ventilação não invasiva e maior permanência na unidade de terapia intensiva associaram-se independentemente à não extubação e à falha de extubação, embora tenham ocorrido apenas quatro falhas.

**Conclusões:**

A extubação na sala operatória foi viável em proporção substancial de pacientes pediátricos e apresentou alta taxa de sucesso. Maior complexidade cirúrgica, necessidade de ventilação não invasiva e maior tempo de permanência na Unidade de Terapia Intensiva (UTI) aumentaram o risco de não extubação, enquanto o aumento da idade o reduziu.

## INTRODUCTION

 Congenital heart diseases are structural defects present at birth. They are a major cause of infant morbidity and mortality worldwide. Cardiac surgery is often essential for the survival of neonates and pediatric patients. This intervention plays a key role in reducing mortality associated with critical cardiac malformations.^1-3^

 Patients undergoing congenital cardiac surgery often require postoperative mechanical ventilation.^4^ Early extubation has become an evidence-based component of enhanced recovery after cardiac surgery protocols, with benefits demonstrated in large, high-volume centers.^5-7^

 Definitions of early extubation vary across studies. Some authors define it as immediate extubation in the operating room. Others define it as extubation within the first six postoperative hours. In neonates, early extubation has also been defined as occurring up to 24 hours after surgery.^8^ Consequently, no universal consensus exists. 

 The American Association for Thoracic Surgery (AATS) Enhanced Recovery consensus recommends immediate operating room extubation for appropriately selected pediatric patients undergoing congenital cardiac surgery as part of an enhanced recovery strategy.^6^ More broadly, the International Clinical Practice Guidelines for Pediatric Ventilator Liberation support protocolized ventilator liberation and timely extubation in critically ill children.^9^ However, these guidelines are intended for the general pediatric population and do not provide specific recommendations regarding the timing or location of extubation after congenital cardiac surgery. Therefore, decisions regarding operating room extubation continue to rely on patient selection, institutional protocols, and multidisciplinary clinical judgment. 

 Several studies report that most patients can be extubated in the operating room.^10-14^ This practice is safe in both simple and complex procedures. Safety has also been demonstrated across different age groups.^15,16^ Immediate extubation is associated with fewer adverse events. It reduces complications related to prolonged mechanical ventilation. It is also associated with shorter stays in intensive care units and hospitals. Collectively, these effects contribute to lower healthcare costs.^17-20^

 In this context, the present study aimed to evaluate the incidence and success rates of intentional postoperative operating room extubation in children submitted to cardiac surgery. In addition, factors independently associated with non-extubation in the operating room and extubation failure were analyzed. 

## METHOD

 This historical cohort study was conducted in a Pediatric and Neonatal Cardiac Intensive Care Unit at a federal referral center for congenital heart disease. The study was approved by the institutional Research Ethics Committee (Certificate of Presentation for Ethical Appreciation — CAAE: 88086425.0.0000.5272). Data were analyzed over five years (January 2020 – December 2024). The study was conducted and reported in accordance with the Strengthening the Reporting of Observational Studies in Epidemiology (STROBE) guidelines for observational studies. 

 The primary outcome was operating room extubation. The secondary outcome was extubation success, defined as the absence of reintubation within the first 48 postoperative hours. Patients were followed from admission to the Pediatric and Neonatal Cardiac Intensive Care Unit until hospital discharge, transfer to another institution, or in-hospital death. 

 Data were obtained from a coded internal database containing hospitalization-related information, which was made available to the multidisciplinary team upon request. The database was complete for all variables included in the analyses, and no imputation procedures were required. 

 A convenience sample was used, including all eligible patients admitted during the study period. Due to the historical design and inclusion of all available cases during the study interval, no a priori sample size calculation was performed. 

 The inclusion criteria comprised patients aged zero to 18 years with congenital heart disease who underwent elective corrective or palliative cardiac surgery and were not receiving preoperative mechanical ventilation. Exclusion criteria were patients undergoing heart transplantation. 

 Patients were classified into two groups: (i) those extubated in the operating room and (ii) those not extubated in the operating room. The extubated group was subsequently subdivided into (i.a) successful extubation and (i.b) unsuccessful extubation. The decision to perform operating room extubation was based on hemodynamic and respiratory stability, as assessed by the anesthesiology team. 

 Collected variables were organized into blocks: sociodemographic (sex and age); anthropometric (weight); clinical (Risk Adjustment for Congenital Heart Surgery — RACHS-1); surgical (palliative or corrective procedure); ventilatory (use and duration of noninvasive ventilation); temporal (length of Intensive Care Unit [ICU] stay); and hospital outcomes (transfer to ward, transfer to another hospital, or death). 

 Descriptive and inferential statistical methods were applied. Categorical variables were expressed as absolute (n) and relative (%) frequencies. Continuous variables were summarized using measures of central tendency and dispersion, including means, medians, and standard deviations. 

 Data distribution was assessed using the Shapiro-Wilk test. Group comparisons were performed according to variable type and distribution, using the chi-square test or Fisher’s exact test for categorical variables, and the Student’s t-test or Mann-Whitney U test for continuous variables. 

 For the multivariable analyses, Poisson regression with robust variance was fitted using non-extubation in the operating room and extubation failure as the modeled outcomes, so that risk ratios (RR) greater than 1 represented increased risk of the adverse outcomes. RRs with 95% confidence intervals (CIs) were calculated. Variables with p<0.05 in univariate analyses were included in the final models. All analyses were conducted using R (version 4.4.2), with a significance level of p≤0.05. 

## RESULTS

 The sample comprised 637 eligible patients, of whom 37% were extubated in the operating room. The extubation success rate was 98%. The patient selection flowchart is shown in Figure 1. 

**Figure 1:**
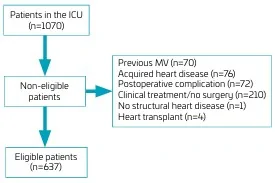
Flowchart of patient selection and study inclusion. Source: The authors, 2026. Abbreviations: ICU: intensive care unit; MV: mechanical ventilation.

 Patients were predominantly male, with a mean age of three years and a mean weight of 13 kg. Most underwent corrective surgery and were classified as RACHS-1 category 2, with transfer to the ward being the most frequent outcome. Detailed baseline characteristics are presented in Table 1. 

**Table 1.** Baseline demographic, clinical, and postoperative characteristics according to operating room extubation status and extubation success.

Variables		Total (n=637)	OR Extubation (n=236)	No OR Extubation (n=401)	Successful (n=232)	Failed (n=4)
Male sex, n (%)		319 (50.1)	120 (50.8)	199 (49.6)	116 (50.0)*	4 (100.0)*
Age, months		39.38±47.40	55.73±47.79	29.73±44.49	56.52±47.80*	9.70±9.91*
Weight, kg		13.14±11.29	16.59±10.79*	11.12±11.09*	16.77±10.80*	6.17±2.69*
RACHS-1, n (%)						
	Category 1	26 (4.1)	9 (3.8)*	17 (4.2)*	9 (3.9)	0 (0.0)
	Category 2	333 (52.3)	152 (64.4)*	181 (45.1)*	150 (64.7)	2 (50.0)
	Category 3	235 (36.9)	74 (31.4)*	161 (40.1)*	72 (31.0)	2 (50.0)
	Category 4	35 (5.5)	1 (0.4)*	34 (8.5)*	1 (0.4)	0 (0.0)
	Category 5	0 (0.0)	0 (0.0)*	0 (0.0)*	0 (0.0)	0 (0.0)
	Category 6	8 (1.2)	0 (0.0)*	8 (2.1)*	0 (0.0)	0 (0.0)
Corrective surgery, n (%)		503 (79.0)	182 (77.1)	321 (80.0)	180 (77.6)	2 (50.0)
Need for NIV, n (%)		122 (19.2)	11 (4.7)	111 (27.7)	8 (3.4)	3 (75.0)
NIV duration, days		5.97±12.21	2.42±1.71*	6.52±13.01*	2.25±1.65	3.33±2.08
ICU length of stay, days		8.29±14.66	4.16±4.93	10.71±17.65	3.92±4.56*	18.00±6.05*
Hospital outcome, n (%)						
	Discharge to ward	586 (92.0)	233 (98.7)*	353 (88.0)*	229 (98.7)	4 (100.0)
	Inter-hospital transfer	21 (3.3)	2 (0.9)*	19 (4.8)*	2 (0.9)	0 (0.0)
	Death	30 (4.7)	1 (0.4)*	29 (7.2)*	1 (0.4)	0 (0.0)

Abbreviations: ICU: intensive care unit; NIV: noninvasive ventilation; OR: operating room; RACHS-1: Risk Adjustment for Congenital Heart Surgery.

Data expressed as mean±standard deviation or n (%). *p-value<0.05.

Source: The authors, 2026.

 Patients extubated in the operating room were, on average, older and had higher body weight than those who were not extubated. This group also had shorter durations of noninvasive ventilation, shorter intensive care unit stays, and lower mortality (Table 1). 

 Similar results were observed when comparing patients with successful and unsuccessful extubation. Notably, all patients with unsuccessful extubation were eventually discharged from the intensive care unit (Table 1). 

 RACHS-1 category 6 was excluded from the multivariable model due to complete separation, since no patients in this category underwent operating room extubation. Multivariable Poisson regression with robust variance estimation demonstrated that each 1-month increase in age was associated with a lower risk of non-extubation in the operating room (RR 0.996), although with a small effect size. Higher surgical complexity increased this risk, particularly the RACHS-1 category 4, which was associated with a 43% increase in the risk of non-extubation (p<0.05). Need for noninvasive ventilation and longer ICU stay were associated with non-extubation in the operating room, increasing the risk by 29 and 0.2% per additional ICU day, respectively. Complete multivariable regression results are shown in Table 2. 

**Table 2.** Factors independently associated with nonextubation in the operating room.

Adjusted variables	RR	95%CI	p-value
Age	0.996	0.994–0.998	<0.001
RACHS-1 Category 2	0.90	0.67–1.20	0.481
RACHS-1 Category 3	1.06	0.80–1.41	0.670
RACHS-1 Category 4	1.43	1.07–1.89	0.014
Need for NIV	1.29	1.16–1.43	<0.001
ICU length of stay	1.002	1.000–1.005	0.035

Abbreviations: RR: risk ratio; CI: confidence interval; RACHS-1: Risk Adjustment for Congenital Heart Surgery; NIV: noninvasive ventilation; ICU: intensive care unit.

Source: The authors, 2026.

 Regarding extubation failure, exploratory analysis suggested an association between the need for noninvasive ventilation and extubation failure; however, these findings should be interpreted cautiously due to their limited precision. Detailed results for extubation failure are presented in Table 3. 

**Table 3.** Factors independently associated with extubation failure.

Adjusted variables	RR	95%CI	p-value
Need for NIV	55.88	5.38–579.98	<0.001
ICU length of stay	1.11	1.07–1.15	<0.001

Abbreviations: RR: risk ratio; CI: confidence interval; NIV: noninvasive ventilation; ICU: intensive care unit.

Source: The authors, 2026.

## DISCUSSION

 This study characterized the epidemiological profile of pediatric patients undergoing congenital cardiac surgery, describing the incidence of operating room extubation and identifying factors independently associated with non-extubation in the operating room and extubation failure. Approximately 37% of patients undergoing cardiac surgery at a federal referral center for congenital heart disease were extubated in the operating room, with a reintubation rate of only 2%. These findings suggest that early extubation is feasible and safe in a substantial proportion of the pediatric cardiac population, consistent with international practices. 

 Reported rates of early extubation range from 27 to 82%, largely reflecting differences in how early extubation is defined. Definitions vary from immediate extubation in the operating room to extubation within the first 24 postoperative hours.^21-27^ Despite this variability, studies consistently demonstrate a trend toward earlier withdrawal of mechanical ventilation in selected patients. 

 The absence of operating room extubation among RACHS-1 category 6 patients corroborates findings by Harris et al., who reported no early extubations in highly complex procedures such as the Norwood operation.^10^ Similarly, none of the eight category 6 patients in the present study were extubated in the operating room. 

 Compared with the results of Tirotta et al., who reported an early extubation rate of 53.1% and a reintubation rate of 1.5%,^13^ the extubation rate observed in this study was lower. One possible explanation is the absence, until 2023, of a structured institutional protocol for operating room extubation selection, highlighting an opportunity for organizational improvement. 

 The absence of a standardized institutional protocol during most of the study period may also have introduced selection bias in extubation practices. In addition, provider- and service-related factors, including the experience of the anesthesiology and surgical teams, competing clinical demands, operating room workflow, and institutional logistics, were not captured in the database. Consequently, these unmeasured variables may have influenced both the decision to perform operating room extubation and postoperative outcomes, introducing residual confounding that could not be addressed in the present analysis. 

 Clinical outcomes demonstrated an association between operating room extubation and a more favorable postoperative evolution. Mortality among patients extubated in the operating room was 0.4%, markedly lower than among those not extubated (7.2%). However, these findings should be interpreted cautiously, as the cohort design of the study does not allow causal inference. It is possible that patients selected for operating room extubation had more favorable intraoperative or postoperative conditions not fully captured in the database, such as shorter cardiopulmonary bypass duration, lower vasoactive support requirements, or better hemodynamic stability. Therefore, operating room extubation may represent not only a therapeutic strategy but also a marker of lower perioperative severity. Nevertheless, the findings are consistent with previous studies reporting associations between prolonged mechanical ventilation and worse postoperative outcomes, including increased mortality.^10^

 Increasing age independently reduced the risk of non-extubation in the operating room (RR 0.996; 95%CI 0.9940.998; p<0.001), suggesting that older children were more likely to undergo immediate postoperative extubation. Previous studies identify age as a relevant prognostic factor, sometimes surpassing weight in predicting mortality after pediatric cardiac surgery.^28^

 Analysis of RACHS-1 categories confirmed the influence of surgical complexity. Category 4 was associated with a 43% higher risk of remaining intubated after surgery (p<0.05). These findings are consistent with Kang et al., who identified RACHS-1 as a predictor of mortality, with a linear relationship between surgical complexity and risk and a nonlinear effect of age.^28^

 Interestingly, although lower surgical complexity is generally expected to favor operating room extubation, RACHS-1 category 1 patients did not demonstrate substantially higher extubation rates compared with category 2 patients. This finding may reflect the multifactorial nature of extubation decisions in pediatric cardiac surgery. Factors such as age, airway characteristics, residual hemodynamic instability, institutional routines, anesthetic management, and individual team preferences may have influenced extubation practices independently of RACHS-1 classification alone. In addition, the relatively small number of category 1 patients may have limited the interpretation of this subgroup. 

 Patients extubated in the operating room also had shorter hospital stays and less frequent use of noninvasive ventilation. The positive association between the need for noninvasive ventilation and extubation failure suggests that the need for postoperative NIV is a marker of greater clinical severity. Likewise, longer ICU stay was independently associated with both non-extubation in the operating room and extubation failure, reinforcing its relationship with greater postoperative severity. 

 Similar results were reported by Romans et al., who observed longer hospital stays, prolonged noninvasive ventilation use, and higher mortality among neonates compared with infants.^29^ Nava et al. also emphasized the role of noninvasive ventilation in ventilatory weaning and ICU length reduction when applied judiciously.^30^

 The findings of the present study should therefore be interpreted within the context of patient selection and institutional practice. Factors associated with a lower risk of non-extubation in the operating room, including older age, were likely markers of greater perioperative stability rather than direct determinants of favorable outcomes. Conversely, greater surgical complexity, the need for noninvasive ventilation, and longer ICU stay were associated with an increased risk of remaining intubated after surgery. These findings suggest that operating room extubation should not be interpreted as an isolated determinant of improved postoperative outcomes, but rather as one component of a broader perioperative recovery strategy applied to appropriately selected patients. 

 This study has limitations inherent to its historical cohort design, including potential selection and information biases. As a single-center study conducted at a federal referral institution for congenital heart disease, the findings may not be fully generalizable to centers with different levels of complexity, surgical expertise, or institutional resources. The lack of standardized extubation criteria may have influenced outcomes. 

 Another limitation is the lack of information on provider- and service-related factors that may have influenced the decision to perform operating room extubation. The database did not include variables such as the experience of the anesthesiology and surgical teams, clinician workload, competing clinical responsibilities, operating room availability, daily surgical volume, or other organizational factors related to operating room workflow. As a result, these unmeasured variables may have influenced both the likelihood of operating room extubation and postoperative outcomes, contributing to residual confounding. 

 Additionally, the absence of detailed intraoperative and hemodynamic variables limits further physiological analyses. No formal sample size calculation was performed; however, the number of events available for the primary outcome was considered adequate for multivariable modelling. In contrast, the low frequency of extubation failure events may have limited the statistical power and stability of the secondary outcome model and should therefore be interpreted with caution. Furthermore, the statistical analysis remains subject to the inherent constraints of the secondary database used. Poisson regression with robust variance was preferred over logistic regression because the primary outcome was relatively frequent, allowing direct estimation of RRs and improving clinical interpretability. 

 In conclusion, operating room extubation was feasible in a substantial proportion of children undergoing elective congenital cardiac surgery and was associated with a high success rate. Older age independently reduced the risk of non-extubation in the operating room, whereas greater surgical complexity, the need for noninvasive ventilation, and longer ICU stay increased the risk of remaining intubated after surgery. Because extubation failure was uncommon, the estimated associations for this outcome should be interpreted cautiously. 

## Data Availability

The database that originated the article is available with the corresponding author.
